# Clinical and genetic analysis of two Chinese families with vitamin D-dependent rickets type IA and follow-up

**DOI:** 10.1186/s13023-020-01558-7

**Published:** 2020-10-01

**Authors:** Yunfei Li, Xin Yuan, Ruimin Chen, Xiangquan Lin, Huakun Shangguan, Xiaohong Yang, Ying Zhang

**Affiliations:** 1 Department of Endocrinology, Genetics and Metabolism, Fuzhou Children’s Hospital of Fujian Medical University, No. 145, 817 Middle Road, Fuzhou, 350005 China; 2 Affiliated Dongfeng General Hospital of Hubei Medical College, Shiyan, 442008 China

**Keywords:** Vitamin D-dependent rickets type IA, *CYP27B1*, Genetic sequence analysis, Treatment, Nephrocalcinosis

## Abstract

**Objective:**

Vitamin D-dependent rickets type IA (VDDR-IA) is a rare autosomal recessive disorder characterized by the early onset of severe rickets. The objectives of this study were twofold: (1) to analyze the clinical characteristics and therapy of two patients with VDDR-IA from two separate Chinese families, and (2) investigate the *CYP27B1* gene mutations in two large pedigrees.

**Methods:**

Medical history, clinical manifestations, physical examination, radiological findings and laboratory data were analyzed from two patients with VDDR-IA. Serum 1, 25-dihydroxyvitamin D [1, 25-(OH)_2_D_3_] of the two patients and their respective families were measured by ELISA and blood samples from both families was obtained for *CYP27B1* gene sequence.

**Results:**

Two patients had typical manifestations and radiological evidence of rickets. Laboratory data showed hypocalcaemia and hypophosphataemia, along with high levels of serum alkaline phosphatase, parathyroid hormone and 25-hydroxyvitamin D_3._ However, serum 1,25-(OH)_2_D_3_ level were low in the patients but normal in their family members. Genetic sequence identified two patients were homozygous for a duplication mutation in exon 8 of *CYP27B1* gene (c.1319_1325dupCCCACCC, p.Phe443Profs * 24). After treating with calcitriol and calcium, there was biochemical improvement with normalization of serum calcium and phosphorus, and radiographic evidence of compensatory skeletal mineralization. One patient developed nephrocalcinosis during follow-up.

**Conclusions:**

This study identified a recurrent seven-nucleotide insertion of *CYP27B1* in two large pedigrees, and compared the clinical characteristics and individual therapy of two affected patients. Additionally, our experience further supports the notion that nephrocalcinosis can occur even on standard doses of calcitriol and oral calcium, and normal level of serum calcium, phosphorus, PTH and 25-(OH)D_3_.

## Introduction

Vitamin D consists of a group of biologically inactive, fat-soluble prohormones which exist in two major forms: ergocalciferol (vitamin D_2_) is mainly produced by plants and cholecalciferol (vitamin D_3_) is derived from 7-dehydrocholesterol in human skin by the action of UV sunlight rays in sunlight [1]. Both forms of vitamin D are activated by a two-step hydroxylation at carbons 25 and 1. The first step occurs in liver, with the production of 25-hydroxyvitamin D_3_ [25-(OH) D_3_] by vitamin D 25-hydroxylase. The second rate-limiting step [2] occurs mainly in kidney proximal tubules where 25-(OH)D_3_ is hydroxylated by the mitochondrial vitamin D1α-hydroxylase, a cytochrome P450 enzyme, to 1, 25-dihydroxyvitamin D_3_ [1, 25-(OH)_2_D_3_], acting through a special vitamin D receptor, the biological actions of 1, 25-(OH)_2_D_3_ encompass the regulation of calcium homeostasis, cellular differentiation, and immune function [1, 3, 4].

Vitamin D-dependent rickets type IA (VDDR-IA), also referred to as pseudo-vitamin D deficiency rickets, was first identified by Prader et al. [5] in 1961. This is a rare autosomal recessive disorder caused by 1α-hydroxylase deficiency. Previous genetic studies have described pathologic mutations in the 25-dihydroxyvitamin D lα-hydroxylase gene (*CYP27B1*), located on 12q13.3 [6, 7]. VDDR-IA is characterized clinically by hypotonia, muscle weakness, growth retardation, hypocalcemic seizures, and skeletal deformities in early infancy, and radiographic findings of rickets with typical laboratory findings of hypocalcemia, elevated serum alkaline phosphatase (ALP) and parathyroid hormone (PTH), normal or increased serum 25-(OH) D_3_ and decreased serum 1, 25-(OH)_2_D_3_ [4]. To date, more than 60 mutations in *CYP27B1* gene have been identified in patients from different ethnic groups [8–12]. These mutations involve all nine exons, and the most common type of *CYP27B1* mutation is a missense mutation, representing more than half of those reports [13].

In the present study, we investigated two patients with VDDR-IA from two separate Chinese families and identified a previously reported mutation of *CYP27B1* gene in two patients and their family members.

### Subjects and methods

This study was approved by the Ethics Committee of the Fuzhou Children's Hospital of Fujian, and informed consent was obtained from the participants’ legal guardians before participating in the study. The two patients are from two Chinese families of Han ethnicity, both of whom lived in Putian City, Fujian Province.

**Fig. 1:**
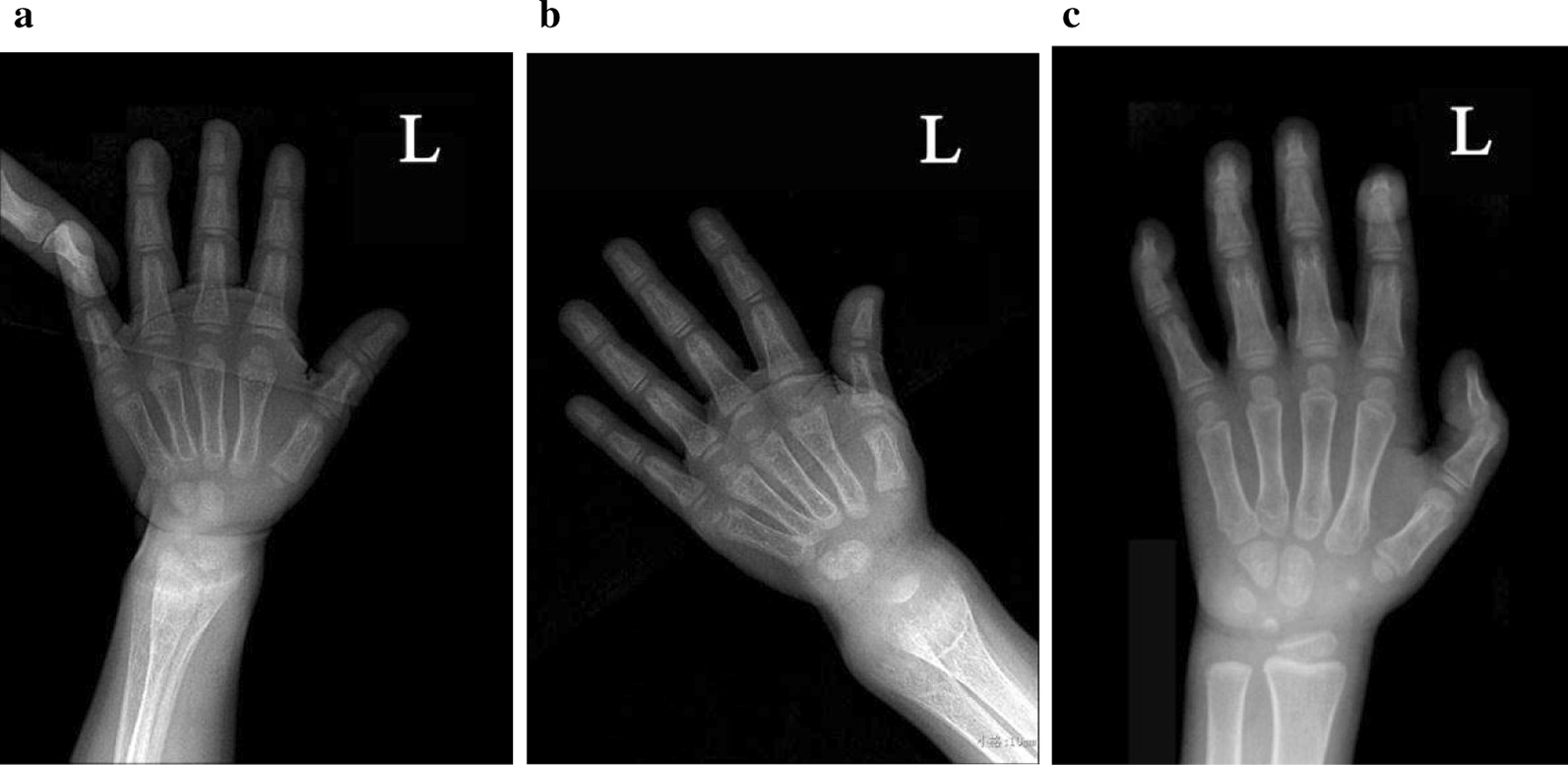
Radiographs of the patient 1 with VDDR-IA. **a** Before treatment, wrist radiograph disclosed the vanishing of the zone of provisional calcification in distal metaphysic, rarefaction of bone trabecula and thinness of the cortical bone in the ulna and radius. **b** After 3 months of treatment, improvement of bone trabecula is obvious. **c** After 1 year of treatment, wrist radiograph showed reoccurrence of the zone of provisional calcification in distal metaphysis, and improvement of bone trabecula and thickening of the cortical

Patient 1 was a 33-month old girl and was the first child of consanguineous parents (first-degree cousins). Her mother’s pregnancy was uneventful. She was born at a full term with a normal birth weight and height. She grew poorly with delayed motor skills, sitting unsupported at the age of 10 months. By 14 months, she could stand with support and had developed a lower limb “O” shaped deformity. She presented with hypocalcaemic seizures at 18 months. Calcitriol 0.25 μg/day and calcium 500 mg/day divided three times a day orally were therapeutic for 15 months. Physical examination showed her height was 76 cm (− 4.84 SD), and weight was 9 kg. Her hair was normal. She had pigeon breast, tibial bowing and widened metaphyses of the wrists and ankles. Laboratory data showed hypocalcemia, hypophosphataemia, low serum 1,25-(OH)_2_D_3_ level, normal serum phosphorus, elevated ALP, 25-(OH)D_3_ and PTH levels (Table 1). Her family members’ biochemical assessment were normal (Table 2). Ultrasonic bone mineral density showed profound osteopenia (− 3.5 SD). X-ray showed typical signs of rickets with widened metaphysic (Fig. 1a).

**Table 1:** Laboratory data of two patients with VDDR-IA

	Ca (mmol/L)	P (mmol/L)	ALP (U/L)	PTH (pg/ml)	25-(OH)D_3_ (ng/ml)	1,25-(OH)_2_D_3_ (pg/ml)
Normal range	2.2–2.8	1.00–1.95	0–500	10–69	15–60	18.7–47.7
Patient 1						
Before treatment	1.36	0.89	1049	235.3	79.5	9.1
After 1 month	1.91	1.38	ND	129	ND	ND
After 3 months	2.2	1.24	ND	246	ND	ND
After 4 months	2.73	1.79	363	59.4	ND	ND
After 1 year	2.63	1.95	ND	22.7	40	ND
After 1.7 year	2.56	1.67	ND	6.54	26.4	ND
After 2 years	2.75	1.17	ND	3	25.2	ND
After 2.5 years	2.64	1.4	ND	3	ND	ND
After 3.3 years	2.43	1.56	270	12.1	34.3	ND
After 3.9 years	2.43	1.71	333	29.6	41.6	ND
After 4.1 years	2.53	1.41	23	ND	37.1	ND
Patient 2						
Before treatment	1.53	0.5	4823	474	149.5	< 5
After 2 months	2.0	0.66	1598	522	ND	ND
After 6 months	1.94	0.83	1540	311	64.37	ND
After 1 year	2.63	1.95	ND	22.7	40	ND
After 1.5 year	2.62	1.6	ND	19	ND	ND
After 2 year	2.56	1.33	361	14.3	28.3	ND
After 2.5 year	2.55	1.32	525	ND	39	ND

*ALP* alkaline phosphatase, *PTH* parathyroid hormone, *ND* not done

**Table 2:** Laboratory data of two families

	Ca (mmol/L)	P (mmol/L)	ALP (U/L)	PTH (pg/ml)	1,25-(OH)_2_D_3_ (pg/ml)
Family 1					
Normal range	2.2–2.8	1.00–1.95	0–500	10–69	18.7–47.7
Father (III 1)	2.6	1.34	78	30.0	41.3
Mother (III 2)	2.57	1.11	57	31.5	39.7
Grandfather (II1)	2.5	1.13	79	35.5	18.9
Grandmother (II 2)	2.41	1.2	90	45.2	21.3
Maternal grandfather (II 3)	2.46	1.1	95	31.4	24.6
Maternal grandmother (II 4)	2.47	1.49	66	53.7	19.3
Great-grandfather (I 1)	2.44	1.74	75	40.7	19.1
Family 2					
Father (III 1)	2.66	1.67	100	22.4	41.3
Mother (III 2)	2.62	1.37	77	21.8	19.7
Grandfather (II 2)	2.64	1.06	79	13.5	19.2
Grandmother (II 1)	2.61	1.68	70	17.8	23.1
Maternal grandfather(II 4)	2.56	1.38	63	14.4	26.6
Maternal grandmother (II 3)	2.48	1.67	62	11.8	27.8
Great-grandfather (I 1)	2.47	1.12	51	21.8	22.8
Great-grandmother (I 2)	2.49	1.34	76	12.3	20.0
Great-grandfather (I 3)	2.57	1.42	132	16.9	34.2
Great-grandmother (I 4)	2.64	1.67	81	11.9	19.4

*ALP* alkaline phosphatase, PTH parathyroid hormone

**Fig. 2:**
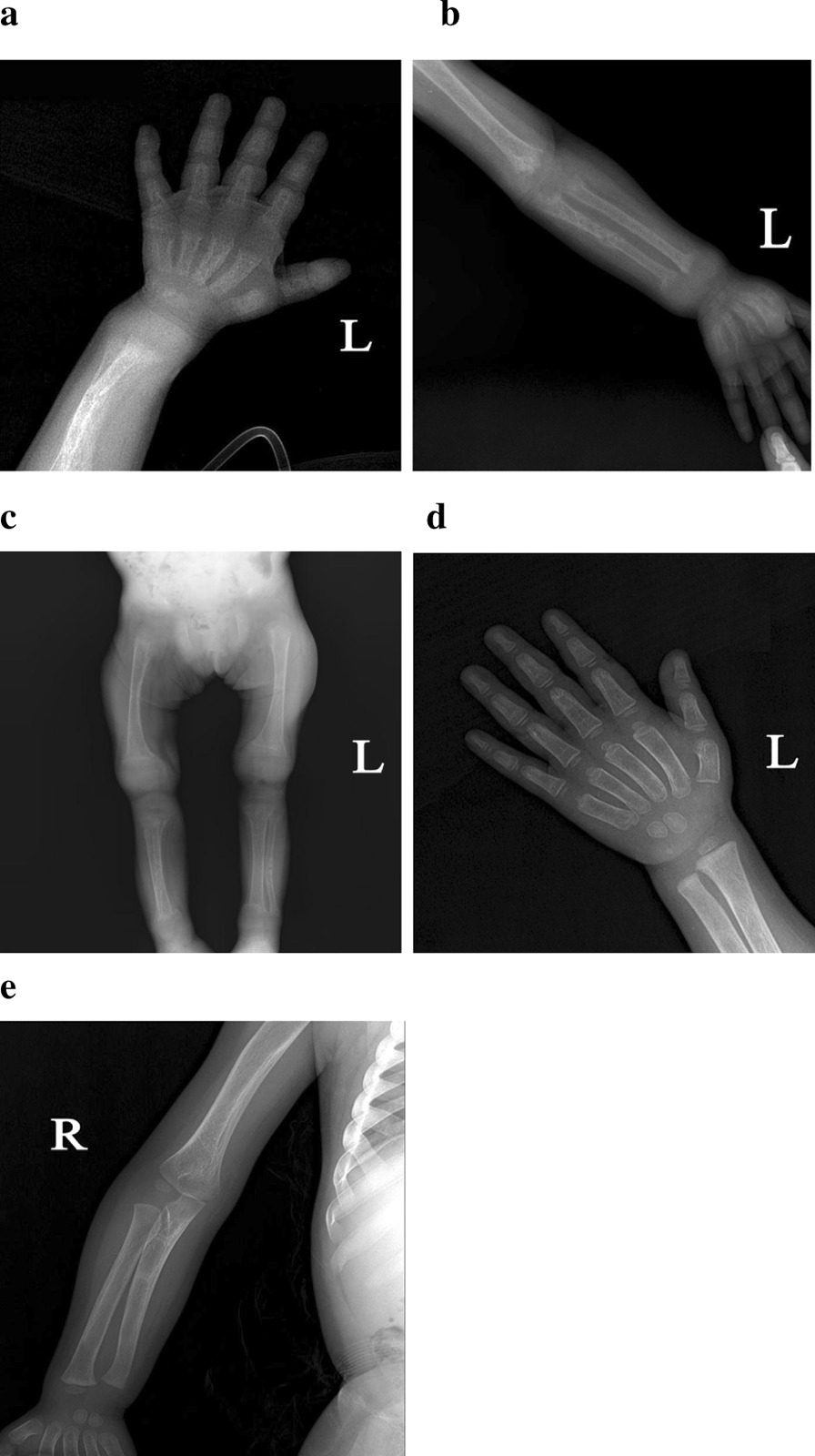
Photographs and radiographs of the patient 2 with VDDR-IA. **a** Before treatment, wrist radiograph disclosed the vanishing of the zone of provisional calcification in distal metaphysic, widened metaphysic and left radius fracture. **b** Before treatment, right ulna fracture with callus formation. **c** Before treatment, lower limbs has thinness of the cortical bone, rarefaction of bone trabecula, widened, frayed, and irregular metaphysis in the long bones, and soft tissue swelling around the wrist joints. **d **After 1 year of treatment, wrist radiograph revealed fracture healing and reoccurrence of the zone of provisional calcification in distal metaphysis, along with improvement of bone trabecula and thickening of the cortical. **e** After 1 year of treatment, right ulna fracture showed healing

Patient 2 was a 22-month old girl and was initially referred to the hospital due to short stature. She was the first child of non-consanguineous parents and was born at full term. Her mother was healthy during pregnancy with no evidence of maternal vitamin D deficiency. She was able to sit unsupported and gradually developed a pigeon breast at 11 months. She suffered from feeding problems (poor appetite and food refusal) and recurrent respiratory infections. She could stand with support, but with evident muscle weakness at 20 months and reportedly irritable. Her height was 68.3 cm (− 5.39 SD), and weight was 7.5 kg. She had late fontanel closure and anterior fontanelle was large (1 cm × 1 cm). She did not have alopecia. There were 18 teeth present and no odontodysplasia. Her maxillary second primary molars were unerupted. Rachitic rosary, eversion of the costal margin, scoliosis and widened metaphyses of the wrists and knees were evident. Laboratory data showed hypocalcemia, hypophosphataemia, low serum 1,25-(OH)_2_D_3_ level, normal serum phosphorus, elevated ALP, 25-(OH)D_3_ and PTH levels (Table 1). Her family members’ biochemical assessment were normal (Table 2). Ultrasonic bone mineral density showed profound osteopenia (− 2.6 SD). X-ray showed osteopenia, widened metaphyses and fractures of right radius and left ulna with callus formation, thinness of the cortical bone, rarefaction of bone trabecula, frayed, and irregular metaphysis in the long bones, consisted with characteristics of rickets (Fig. 2a–c).

### Biochemical assessment

Serum calcium was detected by CPC methods (Reagents: Beijing Leadman Biochemistry Co., Ltd., China; Equipment: Abbott Laboratories C8000, U.S.A.), phosphorus was detected by phosphomolybdate methods (Reagents: Audit Diagnostics, Irish; Equipment: Abbott Laboratories C8000, U.S.A.), ALP was detected by colorimetric method (Reagents: Randox Laboratories Ltd, United Kingdom; Equipment: Abbott Laboratories C8000, U.S.A.), PTH was detected by Chemiluminescence method (SIEMENS IMMULITE 2000 and specific reagents, Germany), 25-(OH)D_3_ were detected by Electrochemical luminescence method (Roche Cobas e411 and specific reagents, Switzerland), and 1,25-(OH)_2_D_3_ were detected by Quantikine Elisa kit (WKSU-BIO, China).

### Imaging techniques

Radiographic studies were performed in the department of radiology at the Fuzhou Children’s Hospital of Fujian Medical University. Plain X-ray of the lower extremities was performed to detect bone deformities.

### DNA sequence analysis of *CYP27B1* gene

After informed consent, peripheral venous blood from both two patients and their family members was taken. In addition, we obtained blood samples from 101 healthy persons of Han ethnicity (37 males, 64 females, mean age 11.3 years) as control, all of whom were long-term residents living in Putian City, Fujian Province.

Genomic DNA from peripheral blood leucocytes was isolated using the QIAamp Blood DNA Mini Kit (TIANGEN, Beijing, China) following the manufacturer’s instructions. All nine exons and intron–exon boundaries of *CYP27B1* were amplified using 2 X PCR MasterMix Taq polymerase (TIANGEN, Beijing, China) by PCR from 100 ng of genomic DNA, and primers were designed as published previously [7]. PCR conditions were 94 °C for 3 min, followed by 30 cycles of amplification (94 °C for 30 s, 55 °C for 30 s, and 72 °C for 1 min). The result of PCR products was directly sequenced using an ABI 3500 DNA sequencer (Life technology, USA). Sequencing results were compared with the *CYP27B1* gene reference sequence (NC_000012.11 and NM_000785.3) to identify the underlying molecular defect VDDR-IA.

### Long-term treatment of calcitriol and calcium

In patients 1 and 2, clinical data was collected before and after treatment with calcitriol and calcium. After starting calcitriol treatment, serum levels of calcium, phosphate, ALP and PTH as well as 25-(OH) D_3_ and 1, 25-(OH)_2_ D_3_ were measured at each clinic visit.

## Results

### Clinical characteristics of the two patients

Both patients presented with failure to thrive, muscle weakness and typical phenotypic features of rickets in early childhood. Laboratory tests showed hypocalcaemia, hypophosphataemia, high levels of serum ALP, PTH and 25-(OH) D_3_, and low serum 1, 25-(OH)_2_D_3_ level. Both patients had normal renal function, which excludes congenital hypophosphatemic rickets characterized by bone mineralization disturbances related to hypophosphatemia secondary to proximal renal loss of phosphate. The diagnosis of 25-hydroxyvitamin D-1α-hydroxylase deficiency was established in each patient based on their clinical and biochemical features.

### Genetic results

Genetic sequence identified two patients were homozygous for a duplication mutation in exon 8 of *CYP27B1* gene (c.1319_1325dupCCCACCC/p.Phe443Profs * 24). No identical mutations were detected in the 101 unrelated control samples (data not shown).

**Fig. 3:**
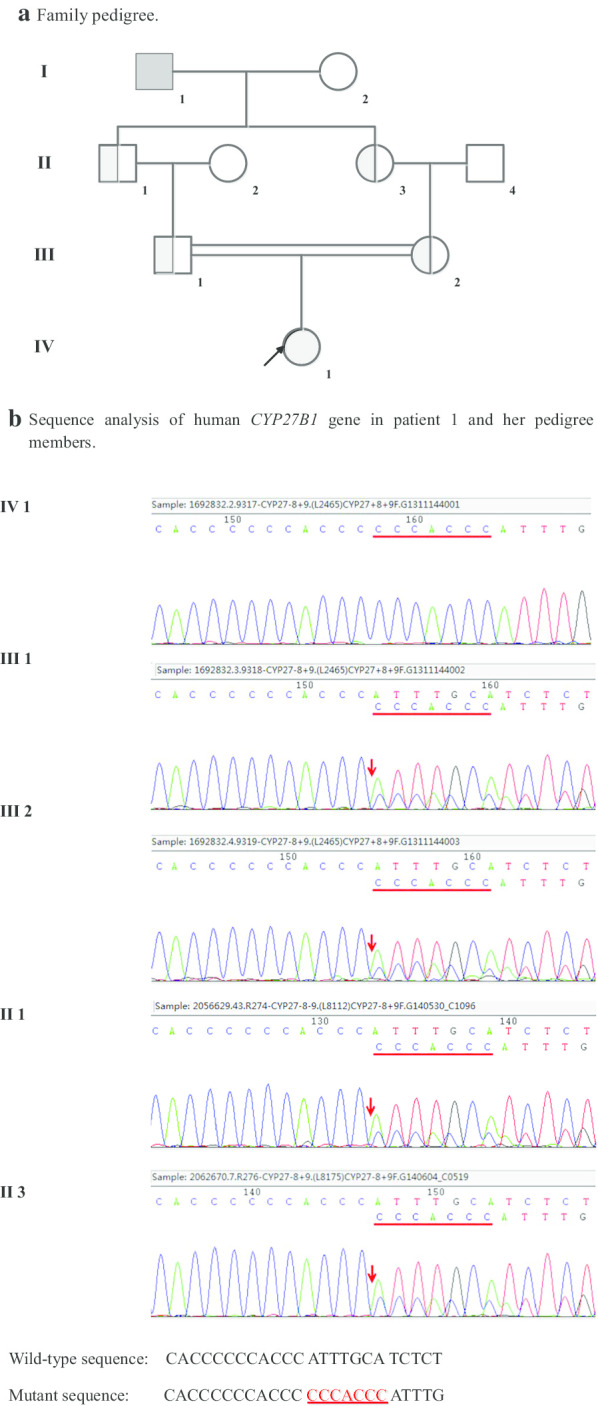
Genetic study of family 1. **a** Family pedigree. Patient 1 is the proband (IV1) of family 1, and the parents (III 1, III 2) are first-degree cousins. The great-grandfather (I1) was not available for genetic evaluation but was predicted to be a carrier. **b** Sequence analysis of human *CYP27B1* gene in patient 1 and her pedigree members. Representative sequence electropherograms are shown. A seven-nucleotide duplication in exon 8 (c.1319_1325dupCCCACCC, p.Phe443Profs * 24) was presented in patient 1 and her father, mother, grandfather and grandmother. Patient 1 was homozygous, others were heterozygous

Family 1 (Fig. 3): The proband (patient 1, IV1) was homozygous for a seven-nucleotide duplication in exon 8 (c.1319_1325dupCCCACCC, p.Phe443Profs * 24). The asymptomatic family members of patient 1, including the father (III 1), the mother (III 2), the grandfather (II 1) and the grandmother (II 3), were all heterozygous carriers. The great-grandfather (I1) was not available for genetic evaluation.

**Fig. 4:**
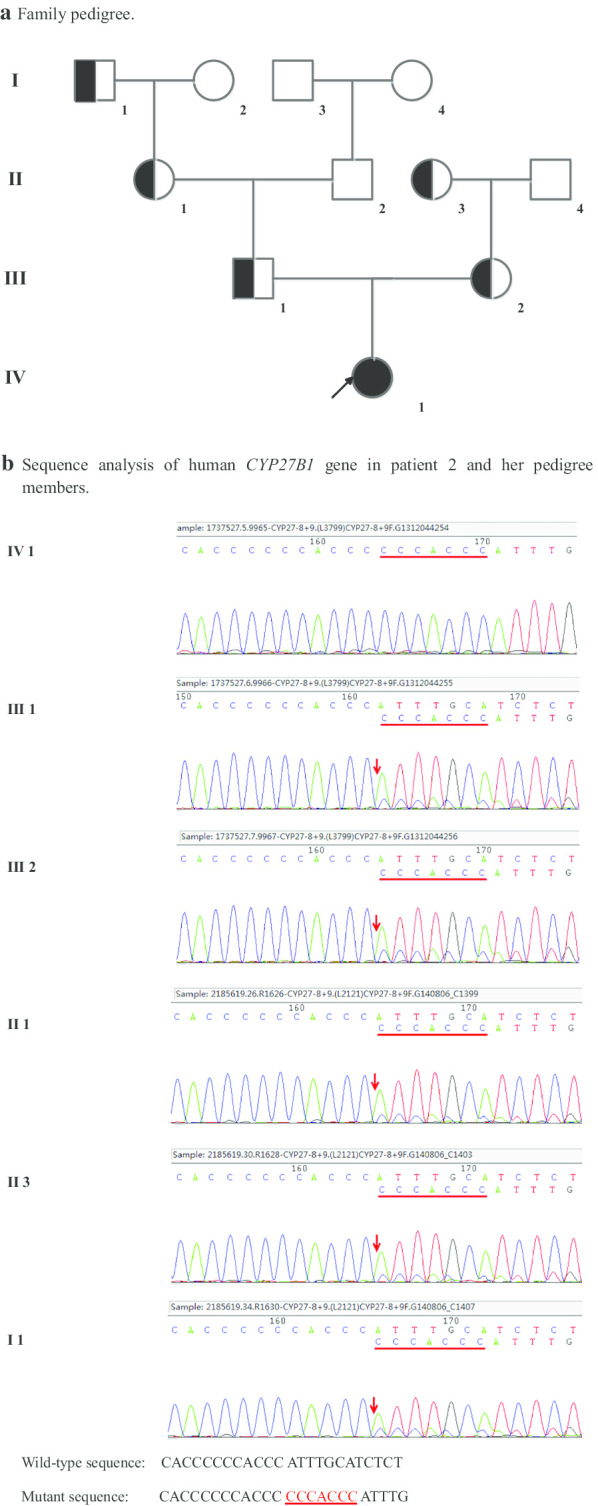
Genetic study of family 2. **a** Family pedigree. Patient 2 is the proband (IV1) of family 2. **b** Sequence analysis of human *CYP27B1* gene in patient 2 and her pedigree members. Representative sequence electropherograms are shown. A seven-nucleotide duplication in exon 8 (c.1319_1325dupCCCACCC, p.Phe443Profs * 24) was presented in patient 2 and her father, mother, grandmother, maternal grandmother and Great-grandfather (I 1). Patient 2 was homozygous, others were heterozygous

Family 2 (Fig. 4): Identical to patient 1, the proband (patient 2, IV1) was homozygous for the seven-nucleotide duplication in exon 8 (c.1319_1325dupCCCACCC, p.Phe443Profs * 24). The father (III 2), mother (III 3), grandmother (II 2), maternal grandmother (II 3) and great-grandfather (I 1) were all heterozygous carriers.

### Clinical outcome after long-term treatment

Patient 1 was initially treated with calcitriol 0.25 μg/day (0.028 μg/kg/day) and calcium 600 mg/day (68 mg/kg/day) orally, divided three times per day. After 1 month of treatment, serum phosphorus normalized with a marked reduction of serum PTH, however, the hypocalcaemia persisted (Table 1). Therefore, calcitriol was increased to 0.75 μg/day (0.079 μg/kg/day) and calcium to 700 mg/day (75 mg/kg/day), divided three times per day. After treatment for 3 months, the calcium returned to normal (Table 1). She was 2.9 cm taller and 0.5 kg heavier than before, and could walk unaided with some gait instability. Improvement of bone trabecula (Fig. 1b) was evident. Over the next month, the serum PTH returned to normal. A year later, the patient demonstrated linear with a growth velocity of 11.2 cm/year (height, 87.2 cm, Z-score: − 3.64 SD), and a catch-up in weight from 9 to 12.2 kg. She ambulated normally. Normalization of serum calcium, phosphorus, PTH and 25-(OH)D_3_ was achieved (Table 1). Wrist radiograph showed reoccurrence of the zone of provisional calcification in distal metaphysis, an improvement in bone trabecula and thickening of the cortical (Fig. 1c). Ongoing treatment insists of calcitriol 1.0 μg/day (0.08 μg/kg/day) and calcium 900 mg/day (78 mg/kg/day) divided three times a day. During the follow-up, kidney ultrasonography has revealed calcareous deposit since treated for 2 years.

Patient 2 was treated with calcitriol 0.75 μg/day (0.1 μg/kg/day) and calcium 300 mg/day (40 mg/kg/day) orally, divided three times a day. After 2 months of treatment, she was 2.7 cm taller and 1 kg heavier. Despite this therapy, hypocalcaemia and hypophosphataemia persisted (Table 1). The doses were increased to calcitriol 1 μg/day (0.12 μg/kg/day) and calcium 700 mg/day (94 mg/kg/day). In the 5th month of treatment, despite an increase in serum calcium and phosphorus, a mild hypocalcaemia and hypophosphataemia persisted (Table 1). Again, the doses were increased to calcitriol 1.5 μg/day (0.18 μg/kg/day) and calcium 900 mg/day (118 mg/kg/day), divided three times a day. After 6 months of supplement, serum calcium returned to normal and she could stand unsupported (Table 1). A year later, she had a growth velocity of 13.7 cm/year (height, 82 cm, Z-score: − 3.25 SD), and a catch-up in weight from 7.5 to 11.1 kg. She could squat and rise unaided. Her serum calcium, phosphorus, PTH and 25-(OH)D_3_ normalized (Table 1). There was resolution of fractures, and improvement in epiphyseal widening with thickening of cortex (Fig. 2d, e). During treatment, kidney function tests showed no abnormalities, and kidney ultrasonography revealed no calculus. She is maintained on calcitriol 2 μg/day (0.18 μg/kg/day) and calcium 1200 mg/day (108 mg/kg/day) divided three times a day.

## Discussion

The causes of rickets include conditions that lead to hypocalcaemia and/or hypophosphatemia, either isolated or secondary to vitamin D deficiency. Disparate etiologies of rickets are: nutritional rickets, hypophosphatemic vitamin D resistant rickets and vitamin D dependent rickets. Neither patients had a medical history of maternal vitamin D deficiency, inadequate sun exposure or inadequate calcium intake, and their increased serum 25-(OH)D_3_ level was inconsistent with nutritional rickets [14]. Hypophosphatemic vitamin D resistant rickets is characterized by copious renal phosphate loss, resulting in elevated urine phosphorus, hypophosphatemia, normal serum levels of calcium, normal or slightly elevated serum PTH and normal or mildly depressed 1, 25-(OH)_2_D_3_ [15, 16]. Our patients had hypophosphatemia and low serum 1, 25-(OH)_2_D_3_ level, but the profound hypocalcaemia is inconsistent with hypophosphatemic vitamin D resistant rickets.

Vitamin D dependent rickets can have two different etiologies. VDDR-IA is caused by 1α-hydroxylase deficiency which impairs the conversion of 25-(OH)D_3_ to 1,25(OH)_2_ D_3_ [17]. It is characterized clinically by early onset of rickets (within the 1st year of life) and severe symptomatic hypocalcemia with concomitant moderate hypophosphatemia [18]. The characteristic biochemical findings of VDDR-IA are normal or increased serum levels of 25-(OH)D_3_ and concomitant low levels of 1, 25-(OH)_2_D_3_ (4). Vitamin D-dependent rickets type II is caused by mutations in the vitamin D receptor gene. Alopecia occurs in approximately two-thirds of cases, consequent to a loss of vitamin D receptor activity within keratinocytes [19]. In stark contrast to VDDR-IA, patients with Vitamin D-dependent rickets type II have elevated concentrations 1, 25-(OH)_2_D_3_.

In our two patients, the aforementioned clinical and biochemical findings were consistent with the VDDR-IA. However, diagnosis should be confirmed by mutational analysis of the *CYP27B1* gene.

Sequence analysis of the *CYP27B1* gene found the same mutation in both patients: homozygous for the seven-nucleotide duplication in exon 8 of *CYP27B1* (c.1319_1325dupCCCACCC, p.Phe443Profs * 24), which is the first report in Chinese patients. Their parents were heterozygous carriers. This mutation changes the reading frame downstream of codon 442 and creates a premature stop signal at codon 466 (Phe443Profs * 24), resulting in complete loss of enzymatic activity. To date, 11 Chinese patients with VDDR-IA have been reported [8, 11, 12, 20]. All patients were compound heterozygous, and 5 of them were compound heterozygous for the seven-nucleotide duplication and one of other mutations.

In 1998, Wang et al. [20] investigated the same mutation in six different ethnic pedigrees, and demonstrated that the protein products of this mutation had no residual 1α-hydroxylase activity. Subsequently, several patients with this mutation were reported in different ethnic groups [9, 21], inferring that this mutation has no apparent ethnic group homology distributions. As controls, we randomly selected 101 healthy people of Han ethnicity from various regions. Sequence analysis of the nucleotide sites (c.1319_1325) in exon 8 of *CYP27B1* gene in this healthy population did not find c.1319_1325dupCCCACCC or other mutations. Hence, this mutation is not a local common variant, rather suggesting a remote consanguinity in these families.

Due to 1α-hydroxylase deficiency, alphacalcidol/calcitriol and calcium supplementation is needed to correct the biochemical abnormalities and skeletal lesions [22]. Both patients responded well to high dose replacement therapy with calcitriol (0.75 μg/day and 2 μg/day or 0.079 μg/kg/day and 0.18 μg/kg/day, respectively). VDDR-IA is can be treated with doses of calcitriol ranging from 0.5 to 2 μg/day or 0.008 to 0.40 μg/kg/day [23]. Durmaz et al. [9] found that calcitriol (0.01–0.1 μg/kg/day) normalized serum calcium and phosphate levels, and promoted growth and development in patients with VDDR-IA. In previously reported Chinese patients, the doses of calcitriol were 0.5–1.0 μg/day [11]. It is noteworthy that patient 2 required higher doses of calcitriol than previously described in Chinese patients. The requisite doses of calcitriol and calcium need to be individualized and sometimes, high-doses of calcitriol are often required. Nephrocalcinosis is uncommon in untreated VDDR-IA but can develop during treatment. So far, only one patient with VDDR-IA developed nephrocalcinosis on oral 0.5 ug/day calcitriol and 750 mg/day calcium [24]. In our experience, patient 1 with normal renal function developed nephrocalcinosis after 2 years of treatment. Additionally, this study corroborates that nephrocalcinosis can occur even on standard doses of calcitriol and oral calcium, and normal level of serum calcium, phosphorus, PTH and 25-(OH)D_3_.

Both of our patients presented with similar features (hypocalcaemic seizure, growth and development retardation, walking difficulty and skeletal deformities) and similar biochemical findings [8, 11, 12, 20]. These common features notwithstanding, the most unusual aspect in our two patients was the severe growth retardation. The underlying molecular mechanism for the severe growth retardation is uncertain. After 1-year follow-up, both linear growth and weight improved. As in our two patients, despite their severe 1, 25-(OH)_2_D_3_ deficiency and concomitant low serum calcium concentrations, bone fractures are not generally a component of VDDR-1A [10, 25].

Durmaz et al. [9] reported one patient with VDDR-IA caused by the *CYP27B1* mutation of c.1319_1325dupCCCACCC. Following calcitriol and treatment, she improved spontaneously at 11 years of age. Interestingly, she did not require treatment to maintain normal serum calcium and 1, 25-(OH)_2_D_3_. Another patient with mild features caused by the same mutation was reported in a series by Wang et al. [20]. Remission might be due to 1a-hydroxylase activity exerted by a non-CYP27B1 enzyme insofar as *CYP27B1* knockout mice can convert 25-(OH)D_3_ to 1, 25-(OH)_2_D_3_ [26].

## Conclusion

In this study, we reported two patients with VDDR-IA with typical clinical manifestations, and genetic sequence analysis found both patients homozygous for the same gene mutation of *CYP27B1*, c.1319_1325dupCCCACCC, p.Phe443Profs * 24. This is the first report of homozygous patients with this seven-nucleotide duplication in Chinese patients, and the first detailed study of the mutation in two large Chinese pedigrees in the same small low-populated city. Furthermore, the comparison and analysis of the clinical characteristics and individual therapy of two patients suggests that high-dose calcitriol is required for those with disturbed severely disturbed calcium homeostasis.

## Data Availability

The datasets during and/or analyzed during the current study are available from the corresponding author on reasonable request.

## Abbreviations

1,25-(OH)2D3: 25-Dihydroxyvitamin D
ALP: Alkaline phosphatase
PTH: Parathyroid hormone
VDDR-IA: Vitamin D-dependent rickets type IA

## References

[1] Holick MF (2007). Vitamin D deficiency. N Engl J Med.

[2] Fu GK, Lin D, Zhang MY, Bikle DD, Shackleton CH (1997). Cloning of human 25-hydroxyvitamin D-1 alpha-hydroxylase and mutations causing vitamin D-dependent rickets type 1. Mol Endocrinol.

[3] Haussler MR, Whitfield GK, Kaneko I, Haussler CA, Hsieh D (2013). Molecular mechanisms of vitamin D action. Calcif Tissue Int.

[4] Miller WL, Portale AA (2003). Vitamin D biosynthesis and vitamin D 1 alpha-hydroxylase deficiency. Endocr Dev.

[5] Prader A, Illig R, Heierli E (1961). An unusual form of primary vitamin D-resistant rickets with hypocalcemia and autosomal-dominant hereditary transmission: hereditary pseudo-deficiency rickets. Helv Paediatr Acta.

[6] Wang X, Zhang MY, Miller WL, Portale AA (2002). Novel gene mutations in patients with 1alpha-hydroxylase deficiency that confer partial enzyme activity in vitro. J Clin Endocrinol Metab.

[7] Alzahrani AS, Zou M, Baitei EY, Alshaikh OM, Al-Rijjal RA (2010). A novel G102E mutation of CYP27B1 in a large family with vitamin D-dependent rickets type 1. J Clin Endocrinol Metab.

[8] Cao L, Liu F, Wang Y, Ma J, Wang S (2011). Novel vitamin D 1alpha-hydroxylase gene mutations in a Chinese vitamin-D-dependent rickets type I patient. J Genet.

[9] Durmaz E, Zou M, Al-Rijjal RA, Bircan I, Akçurin S, Meyer B, Shi Y (2012). Clinical and genetic analysis of patients with vitamin D-dependent rickets type 1A. Clin Endocrinol (Oxf).

[10] Babiker AM, Al Gadi I, Al-Jurayyan NA, Al Nemri AM, Al Haboob AA, Al Boukai AA, Al Zahrani A, Habib HA (2014). A novel pathogenic mutation of the CYP27B1 gene in a patient with vitamin D-dependent rickets type 1: a case report. BMC Res Notes.

[11] Cui N, Xia W, Su H, Pang L, Jiang Y, Sun Y (2012). Novel mutations of CYP27B1 gene lead to reduced activity of 1alpha-hydroxylase in Chinese patients. Bone.

[12] Cui N, Xia W, Su H, Pang L, Jiang Y, Sun Y, Nie M, Xing X, Li M, Wang O, Yuan T, Chi Y, Hu Y, Liu H, Meng X, Zhou X (2014). A novel compound mutation of CYP27B1 in a Chinese family with vitamin D-dependent rickets type 1A. J Pediatr Endocrinol Metab.

[13] Sawada N, Sakaki T, Kitanaka S, Kato S, Inouye K (2001). Structure-function analysis of CYP27B1 and CYP27A1. Studies on mutants from patients with vitamin D-dependent rickets type I (VDDR-I) and cerebrotendinous xanthomatosis (CTX). Eur J Biochem.

[14] Donghi V, Di Frenna M, di Lascio A, Chiumello G, Weber G (2011). Vitamin D dependent rickets, diagnostic and therapeutic difficulties: two case reports. J Pediatr Endocrinol Metab.

[15] Brame LA, White KE, Econs MJ (2004). Renal phosphate wasting disorders: clinical features and pathogenesis. Semin Nephrol.

[16] Yuan B, Xing Y, Horst RL, Drezner MK (2004). Evidence for abnormal translational regulation of renal 25-hydroxyvitamin D-1alpha-hydroxylase activity in the hyp-mouse. Endocrinology.

[17] Kitanaka S, Takeyama K, Murayama A, Kato S (2001). The molecular basis of vitamin D-dependent rickets type I. Endocr J.

[18] Sahay M, Sahay R (2012). Rickets-vitamin D deficiency and dependency. Indian J Endocrinol Metab.

[19] Sahay M, Sahay R (2012). Renal rickets-practical approach. Indian J Endocrinol Metab.

[20] Wang JT, Lin CJ, Burridge SM, Fu GK, Labuda M, Portale AA, Miller WL (1998). Genetics of vitamin D 1alpha-hydroxylase deficiency in 17 families. Am J Hum Genet.

[21] Ito N, Peña AS, Perano S, Atkins GJ, Findlay DM, Couper JJ (2014). First Australian report of vitamin D-dependent rickets type I. Med J Aust.

[22] Labuda M, Fujiwara TM, Ross MV, Morgan K, Garcia-Heras J, Ledbetter DH, Hughes MR, Glorieux FH (1992). Two hereditary defects related to vitamin D metabolism map to the same region of human chromosome 12q13-14. J Bone Miner Res.

[23] Delvin EE, Glorieux FH, Marie PJ, Pettifor JM (1981). Vitamin D dependency: replacement therapy with calcitriol?. J Pediatr.

[24] Laway BA, Wani AI, Masoodi SR, Bashir MI, Ganie MA, Zargar AH (2010). Hypercalciuria and nephrolithiasis on long-term follow-up of pseudo-vitamin D deficiency rickets. J Pak Med Assoc.

[25] Yan Y, Calikoglu AS, Jain N (2011). Vitamin D-dependent rickets type 1: a rare, but treatable, cause of severe hypotonia in infancy. J Child Neurol.

[26] Rowling MJ, Gliniak C, Welsh J, Fleet JC (2007). High dietary vitamin D prevents hypocalcemia and osteomalacia in CYP27B1 knockout mice. J Nutr.

